# Ocular Clinical Signs and Diagnostic Tests Most Compatible With Keratoconjunctivitis Sicca: A Latent Class Approach

**DOI:** 10.1097/ICO.0000000000002311

**Published:** 2020-08

**Authors:** John A. Gonzales, Stephen C. Shiboski, Vatinee Y. Bunya, Esen K. Akpek, Jennifer Rose-Nussbaumer, Gerami D. Seitzman, Lindsey A. Criswell, Caroline H. Shiboski, Thomas M. Lietman

**Affiliations:** * Department of Ophthalmology, Francis I. Proctor Foundation, University of California San Francisco, San Francisco, CA;; † Department of Ophthalmology, University of California San Francisco, San Francisco, California;; ‡ Department of Epidemiology and Biostatistics, University of California San Francisco, San Francisco, CA;; § Department of Ophthalmology, Scheie Eye Institute, University of Pennsylvania, Philadelphia, PA;; ¶ Ocular Surface Diseases and Dry Eye Clinic, The Wilmer Eye Institute, Johns Hopkins University, Baltimore, MD;; ǁ The Johns Hopkins Jerome L. Greene Sjögren’s Syndrome Center,k Baltimore, MD;; ** Department of Ophthalmology, Kaiser Permanente, Redwood City, CA;; †† Departments of Medicine; and Orofocial Sciences, School of Dentistry, University of California San Francisco, San Francisco, CA.; ‡‡ Department of Orofocial Sciences, School of Dentistry, University of California San Francisco, San Francisco, CA.

**Keywords:** keratoconjunctivitis sicca, Sjogren syndrome, tear breakup time, ocular staining score, Schirmer 1, tear osmolarity, Lissamine Green, fluorescein

## Abstract

**Purpose:**

To evaluate the ocular signs and tests for keratoconjunctivitis sicca (KCS) in the absence of a gold standard.

**Methods:**

Cross-sectional study of participants from the Sjögren’s International Collaborative Clinical Alliance (SICCA) registry. Participants had oral/ocular/rheumatologic examinations, blood/ saliva samples collected, and salivary gland biopsy. Latent class analysis (LCA) identified clusters of patients based on 3 to 4 predictor variables relating to signs or tests of KCS. The resulting model-based “gold standard” classification formed the basis for estimated sensitivity and specificity associated with these predictors.

**Results:**

A total of 3514 participants were enrolled into SICCA, with 52.9% classified as SS. LCA revealed a best-fit model with 2 groups. For the gold standard–positive group, an abnormal tear breakup time, ocular staining score (OSS), and Schirmer I had a sensitivity of 99.5%, 91.0%, and 47.4%, respectively. For the gold standard–negative group, an abnormal tear breakup time, OSS, and Schirmer I had a specificity of 32.0%, 84.0%, and 88.5%, respectively. OSS components (fluorescein and lissamine staining), exhibited a sensitivity of 82.6% and 90.5%, respectively, in the gold standard–positive group, whereas these signs in the gold standard–negative group had a specificity of 88.8% and 73.0%, respectively.

**Conclusions:**

OSS and its components (fluorescein and lissamine staining) differentiated 2 groups from each other better than other KCS parameters and had relatively high sensitivity and specificity.

Sjogren syndrome is a common autoimmune disease best known for causing dry eye and dry mouth but also features more widespread extraglandular manifestations including neuropathy, renal disease, and systemic vasculitis.^1–5^ Dry eye is a hallmark feature of Sjogren syndrome (SS), yet SS is underdiagnosed in patients with dry eye disease.^6–9^ When Henrik Sjögren described a specific type of dry eye, that of keratoconjunctivitis sicca (KCS), he highlighted its features of corneal and conjunctival staining with rose bengal.^10–12^ Other measures for identifying dry eye include Schirmer testing and newer methods including assessing tear osmolarity and inferior tear meniscus height.^13–21^ Use of any diagnostic test or sign should accurately differentiate between patients with a disease state and those without. For dry eye, ideally there would be a single test that would serve as the reference or gold standard. In reality, however, no such gold standard exists.

Instead, there are a variety of ocular signs and tests used to identify dry eye either alone or in combination with each other. These measures include the assessment of corneal staining with fluorescein, conjunctival staining with lissamine green, the ocular staining score (OSS), tear breakup time (TBUT), Schirmer testing, and tear osmolarity.^22^ In addition, patient-reported symptoms of dry eye are included in the assessments for identifying patients with dry eye disease.^23,24^ By using different reference standards, the classification of patients with dry eye may be haphazard between different studies. As a result, the quantitative assessment of the performance of different index tests and estimates of disease prevalence may be biased.^25^

In this study, we applied latent class analysis (LCA) to data from participants in the Sjögren’s International Collaborative Clinical Alliance (SICCA) to estimate the sensitivity and specificity of some of the most commonly used clinical signs and tests to diagnose KCS. We used the term KCS intentionally because we applied LCA to diagnostic tests and signs to the SICCA cohort, which was constructed, in part, to assess the relationship of aqueous deficiency to Sjogren syndrome. In the absence of a gold standard KCS classification, this model-based approach provides a preliminary sense of relative diagnostic performance of these measures.

## METHODS

### Study Design and Population

The SICCA cohort represents a cross-sectional study of participants enrolled from 9 international research sites. Participants (≥21 years of age) met at least one of the following conditions: 1) complaint of dry eye or dry mouth, 2) previous diagnosis of primary or secondary Sjogren syndrome (SS), 3) abnormal serology (positive anti-SSA, anti-SSB, or elevated ANA and RF), 4) bilateral parotid gland enlargement, or 5) multiple cervical/incisal dental caries.

At the baseline SICCA visit, participants had completed an interview and questionnaires, had oral/ocular/rheumatologic examinations, had blood and saliva samples collected, and had a labial salivary gland biopsy among other procedures.^26,27^ Ethical clearance was obtained from the institutional review board at each clinical site, and the study adhered to the tenets of the Declaration of Helsinki.

### Variables and Measures

Independent variables that were recorded included OSS (abnormal ≥ 5), Schirmer I (abnormal ≤ 5 mm in 5 minutes), TBUT (abnormal < 10 seconds), and tear osmolarity (abnormal > 316 mOsm/L) as measured with the TearLabTM system (TearLab, San Diego, CA).

### Statistical Analysis

*Levels of sensitivity and specificity for each relevant clinical sign and* diagnostic test (variable) were estimated using LCA. This analysis provides a model-based clustering of participants into a specified number of “disease” classes based on the observed patterns of a series of binary predictor variables representing the presence or absence of important diagnostic features. The resulting classes can then be related to the disease status based on the class-specific patterns of diagnostic features. Our modeling was based on 4 or 5 predictor variables that relate to signs of KCS. Models incorporating different numbers of classes were compared using the Bayes information criterion (BIC). In addition, we also fitted the LCA models incorporating a random effect to relax the conventional assumption of conditional independence of within-class outcomes. Finally, sensitivity and specificity of individual predictors were estimated using the model-based classification with an underlying, unobservable “gold standard.^26,28,29^ Analyses were performed with the R package randomLCA (R Core Team, R version 3.3.2 and R Studio 1.0.136, R, Boston, MA).

## RESULTS

A total of 3514 participants from 9 international sites were enrolled in SICCA. Most participants were women [3185 (91%) women vs. 309 (9%) men]. Gender was missing for 20 participants. Sjogren syndrome as defined by the ACR/ EULAR criteria was diagnosed in 1541 participants (52.9%) and 116 participants (3.3%) could not be classified because of the missing data.

With LCA, we found a best-fit model with 2 groups. Incorporation of a random effect did not noticeably improve the model fit. The gold standard–positive group having an abnormal TBUT, OSS, and Schirmer I had a sensitivity of 99.5% (95% CI: 94.9%–100%), 91.0% (95% CI: 84.2%–95.1%), and 47.4% (95% CI: 44.6%–50.4%), respectively (Table 1). The gold standard–negative group having an abnormal TBUT, OSS, and Schirmer I had a specificity of 32.0% (95% CI: 28.6%–35.5%), 84.0% (95% CI: 79.0%–87.9%), and 88.5% (95% CI: 85.0%–91.2%), respectively (Table 1).

Only 79 participants had tear osmolarity tested because of the late addition of this test during the study period. When we included tear osmolarity in the model, we found that the gold standard positive–group having an abnormal TBUT, OSS, Schirmer I, and osmolarity had a sensitivity of 99.5% (95% CI: 94.9%–100%), 91.1% (95% CI: 84.2%–95.1%), 47.5% (95% CI: 44.6%–50.4%), and 45.7% (95% CI: 29.6%–62.7%), respectively (Table 2). The gold standard–negative group having an abnormal TBUT, OSS, Schirmer I, and osmolarity had a specificity of 32.0% (95% CI: 28.6%–35.5%), 83.9% (95% CI: 79.0%–87.9%), 88.5% (95% CI: 85.0%–91.2%), and 72.1% (95% CI: 53.2%–85.4%), respectively (Table 2).

Because the OSS comprises corneal staining with fluorescein and conjunctival staining with lissamine green, we wished to determine whether staining of the cornea or conjunctiva was able to distinguish the 2 groups from each other. LCA revealed that for the gold standard–positive group having an abnormal TBUT, fluorescein, lissamine, and Schirmer I had a sensitivity of 98.9% (95% CI: 97.7%–100%), 82.6% (95% CI: 78.9%–85.7%), 90.5% (95% CI: 87.2%–93.0%), and 49.8% (95% CI: 46.7%–52.9%), respectively (Table 3). In the gold standard–negative group, the specificity for an abnormal TBUT, fluorescein, lissamine, and Schirmer I was 29.1% (95% CI: 26.5%–31.7%), 88.8% (95% CI: 85.3%–91.6%), 73.0% (95% CI: 69.6%–76.2%), and 88.1% (95% CI: 86.1%–89.8%), respectively (Table 3).

## DISCUSSION

By using LCA, we inferred that both TBUT (<10 seconds) and OSS (≥5) had a high sensitivity in identifying 2 groups of individuals within the population of patients with dry eye in the SICCA cohort—“dry” and “not dry”. However, although OSS exhibited a high specificity, TBUT did not. We found that the OSS’s constituent components (lissamine green staining of the temporal and bulbar conjunctiva and fluorescein staining of the cornea) also exhibited high sensitivity and high specificity. Tear osmolarity, on the other hand, was neither sensitive nor particularly specific in distinguishing between 2 groups of patients with dry eye, although few participants in this cohort had tear osmolarity tested, which prompted us to include this variable as a sensitivity analysis. Although Schirmer I testing was not particularly sensitive, it was specific. Although Schirmer I has been criticized previously for exhibiting variability,^23,30,31^ the Schirmer I should be considered as complementary when combined with the use of the OSS. Taken together, our results suggest that the OSS or its constituent components are particularly sensitive and specific in identifying participants with keratoconjunctivitis sicca in our cohort. In addition, Schirmer I testing is complementary in that it is a specific test that can corroborate aqueous deficiency that typifies lacrimal gland dysfunction in Sjogren syndrome.

Currently, the application of classification criteria for dry eye disease varies depending on the study, making comparisons difficult. It is essential to have classification criteria for KCS for clinical trials and epidemiological studies to provide internal study consistency and to allow for comparisons between studies.

The OSS is composed of 2 types of stains used to identify the desiccated areas of the cornea or bulbar conjunctiva. Because concentrated lissamine green 1% solution is not commercially available, it must be prepared either by a specialty compounding pharmacy (which is not always available or an option at some centers because of differing compounding laws) or may be too time consuming to make for each patient, although protocols for making it using lissamine green–impregnated strips exist.^32^ Using components of the OSS (fluorescein and lissamine green staining) yielded a relatively high sensitivity for corneal fluorescein staining using a cutoff of 80%. Therefore, the assessment of corneal fluorescein staining alone may be a useful way to identify patients with KCS. This is reassuring because fluorescein strips are readily available and used in most ophthalmology clinics. In addition, this study shows that other tests, such as Schirmer I, can be complementary when combined with the OSS or with fluorescein staining alone when identifying patients with KCS.

In contrast to our results, independent component analysis (ICA) was used to suggest that tear osmolarity might be the best marker for identifying dry eye severity.^33^ ICA is a signal processing technique in which signals are assumed to be statistically independent.^34^ If used for diagnostic test studies, ICA would reward tests that are uncorrelated to other tests. Although tear osmolarity was identified as independent— that does not imply importance—a random number generator would have been identified as even more independent and would presumably play no role in dry eye detection.^35^ In one study, mean osmolarity of patients with blepharitis and SS was higher than in controls, although not significantly so.^20^ Some have suggested that corneal and conjunctival staining are inherently insensitive, but our findings suggest quite the opposite.^36^ We do stress the importance, however, of using concentrated lissamine green 1% in staining the conjunctiva because using single lissamine green–impregnated strip on conjunctiva does not adequately stain.^32^

A limitation of this study, similar to other studies of keratoconjunctivitis sicca is the use of expert clinical judgment in the absence of an objective “gold standard” for defining keratoconjunctivitis sicca. However, although expert opinion has driven the use of dry eye clinical signs and tests including conjunctival staining, corneal staining, Schirmer I testing, TBUT, and tear osmolarity, we applied a latent class model-based approach to allow us to cluster SICCA participants for keratoconjunctivitis sicca. Limitations of LCA include its unsupervised approach. In addition, LCA makes assumptions; in particular, it assumes that each diagnostic test is conditionally independent. Nevertheless, LCA can be a robust statistical method that analyses categorically-scored data particularly when 3 or more diagnostic tests or signs are being evaluated as in the present study’s case.

Using the uniform diagnostic criteria for KCS may be useful for future studies of Sjogren syndrome. One of the contributions of SICCA has been the development of standardized diagnostic criteria for SS. Our analyses presented herein demonstrate that the OSS, which harkens back to Henrik Sjögren’s original description of keratoconjunctivitis sicca, is a hallmark and distinguishing feature of aqueous deficiency in his eponymous syndrome.

## Figures and Tables

**TABLE 1.** Sensitivity and Specificity for Each Keratoconjunctivitis Sicca Clinical Sign or Test in the Entire Cohort

Ocular Clinical Sign or Test	Sensitivity (95% CI)	Specificity (95% CI)
TBUT < 10 s	99.5% (94.9%–100%)	32.0% (28.6%–35%)
OSS ≥ 5	91.0% (84.2%–95.1%)	84.0% (79.0%–87.9%)
Schirmer I ≤ 5 mm/5 min	47.4% (44.6%–50.4%)	88.5% (85.0%–91.2%)

**TABLE 2.** Sensitivity and Specificity for Each Keratoconjunctivitis Sicca Clinical Sign or Test in the Entire Cohort Including Tear Osmolarity

Ocular Clinical Sign or Test	Sensitivity (95% CI)	Specificity (95% CI)
TBUT < 10 s	99.5% (94.9%–100%)	32.0% (28.6%–35.5%)
OSS ≥ 5	91.1% (84.2%–95.1%)	83.9% (79.0%–87.9%)
Schirmer I ≤ 5 mm/5 min	47.5% (44.6%–50.4%)	88.5% (85.0%–91.2%)
Osmolarity*	45.7% (29.6%–62.7%)	72.1% (53.2%–85.4%)

* Seventy-nine participants had tear osmolarity tested.

**TABLE 3.** Sensitivity and Specificity for Each Keratoconjunctivitis Sicca Clinical Sign or Test in the Entire Cohort With OSS Separated Into Its Components, Lissamine and Fluorescein

Ocular Clinical Sign or Test	Sensitivity (95% CI)	Specificity (95% CI)
TBUT < 10 s	98.9% (97.7%–99.5%)	29.1% (26.5%–31.7%)
Fluorescein ≥ grade 3	82.6% (78.9%–85.7%)	88.8% (85.3%–91.6%)
Lissamine ≥ grade 3	90.5% (87.2%–93.0%)	73.0% (69.6%–76.2%)
Schirmer I ≤ 5 mm/5 min	49.8% (46.7%–52.9%)	88.1% (86.1%–89.8%)
